# Ubiquitin-specific peptide 22 acts as an oncogene in gastric cancer in a son of sevenless 1-dependent manner

**DOI:** 10.1186/s12935-020-1137-y

**Published:** 2020-02-10

**Authors:** ChitChoon Lim, Jia-Cheng Xu, Tian-Yin Chen, Jia-Xin Xu, Wei-Feng Chen, Jian-Wei Hu, Quan-Lin Li, Yi-Qun Zhang

**Affiliations:** Endoscopy Center and Endoscopy Research Institute, Zhongshan Hospital, Fudan University, No. 180 FengLin Road, Shanghai, 200032 China

**Keywords:** Ubiquitin-specific peptide 22, Son of sevenless 1, RAS protein, Gastric cancer

## Abstract

**Background:**

Aberrant expression of ubiquitin-specific peptide 22 (USP22) has been detected in various cancers. This study aimed to investigate the role of USP22 and the underlying mechanism in human gastric cancer.

**Methods:**

The expression pattern of USP22 in human gastric cancer was detected in a tissue microarray containing 88 pairs of gastric cancer tissue and adjacent normal tissue samples from patients with primary gastric cancer using immunohistochemical staining. The correlation of USP22 expression with clinical characteristics of patients, as well as their prognostic values in the overall survival of patients, were evaluated. USP22-overexpressing SGC7901 and USP22-silencing AGS cells were used to explore the role of USP22 in gastric cancer cell behavior in vitro and in vivo. Chromatin immunoprecipitation was performed to identify differentially expressed genes induced by USP22 overexpression. Western blot analysis was conducted to detect the activation of RAS/ERK and PI3K/AKT signaling in USP22-overexpressing SGC7901 cells and xenograft tumor tissues. Knockdown of RAS activator son of sevenless 1 (SOS1) was performed to investigate the role of SOS1 in USP22-regulated gastric cancer cell behavior and RAS signaling both in vitro and in vivo.

**Results:**

USP22 protein expression was significantly increased in human gastric cancer tissues, compared with adjacent normal tissues, and was positively correlated with local tumor stage. Gain- and loss-of-function assays showed that USP22 promoted gastric cancer cell growth and cell cycle transition while suppressing apoptosis in vitro. Consistent results were observed in a xenograft mouse model. Chromatin immunoprecipitation revealed that the overexpression of USP22 induced the upregulation of RAS activator son of sevenless 1 (SOS1) in SGC7901 cells. Western blot analysis showed that USP22 overexpression also induced activation of the RAS/ERK and PI3K/AKT pathways in SGC7901 cells and xenograft tumor tissues. Furthermore, SOS1 silencing could reverse the effects of USP22 on gastric cancer cell behavior and RAS signaling both in vitro and in vivo.

**Conclusions:**

Our results suggest that USP22 acts as an oncogene in gastric cancer in a SOS1-dependent manner, identifying the USP22/SOS1/RAS axis as a potential therapeutic target in gastric cancer.

## Background

Gastric cancer is the fifth most commonly diagnosed cancer and the third leading cause of cancer death worldwide, responsible for over 1 million new cancer cases in 2018 [1]. In China, there are approximately 400,000 new cases of gastric cancer per year, 80% of which are diagnosed at an advanced stage [2]. It is important to reveal the mechanisms underlying gastric cancer development and to identify promising therapeutic targets for gastric cancer therapy.

Ubiquitin-specific peptide 22 (USP22) is a deubiquitinating enzyme that participates in the formation of a transcriptional regulatory histone acetylation complex. USP22 removes ubiquitin from histones H2A and H2B, thereby modulating the transcription of target genes [3, 4]. USP22 can also stabilize target proteins by protecting them from proteasomal degradation through deubiquitination [5]. USP22 overexpression occurs in many cancer types, including gastric cancer [6], playing oncogenic roles via multiple mechanisms, such as the activation of transcription factors [5], the attenuation of cancer cell apoptosis [7], and promotion of the cell cycle transition [8]. Targeting USP22 and its downstream effectors is a promising strategy in cancer therapy. However, recent studies have challenged the tumorigenic properties of USP22 in cancer. Gene sequencing data have revealed that USP22 mRNA expression is frequently reduced in some cancers, including ovarian, esophageal, and stomach cancers [9]. Kosinsky et al. found that USP22 exerts tumor-suppressive functions in colorectal cancer by decreasing mTOR activity [10]. Therefore, clarifying the roles of USP22 in cancer is critical to determine whether direct USP22 targeting will be beneficial for patients.

The aberrant activation of RAS proteins plays a causal role in human cancer [11]. Son of sevenless 1 (SOS1) is a guanine nucleotide exchange factor (GEF) that catalyzes the formation of active, GTP-bound RAS, which triggers a wide range of downstream signaling pathways [12]. Targeting GEFs such as SOS1 represents a strategy to reduce the level of active RAS in RAS-driven tumors [13, 14]. Extracellular signal-regulated kinase (ERK) and phosphatidylinositol 3-kinase (PI3K) are two principal effectors of RAS [15]. Aberrant activation of the RAS/ERK and RAS/PI3K/AKT pathways has been observed in gastric cancer [16–18]. RAS mutations are often responsible for RAS overactivation in human cancers [19]; however, gastric cancer cells rarely carry RAS mutations, suggesting that alternative mechanisms contribute to RAS overactivation in this type of cancer [16]. Although previous studies have identified many genetic alterations, epigenetic modifications, and environmental factors that are responsible for hyperactive RAS in cancer [16], the role of SOS1/RAS signaling in gastric cancer remains unknown.

In this study, we measured the protein expression of USP22 in gastric cancer tissue in patients with primary gastric cancer and evaluated the correlation between USP22 expression and clinicopathologic characteristics. We also investigated the effects of USP22 overexpression and knockdown on the malignant behavior of gastric cancer cells in vitro and in vivo. Furthermore, our study indicated that SOS1 was upregulated in USP22-overexpressing gastric cancer cells and xenograft tumor tissue, accompanied by activation of the RAS/ERK and PI3K/AKT pathways, suggesting that SOS1/RAS signaling mediates the oncogenic role of USP22 in gastric cancer. Our findings identify the USP22/SOS1/RAS axis as a promising therapeutic target in gastric cancer.

## Methods

### Patients and specimens

Gastric cancer tissue and corresponding adjacent normal tissue (> 5 cm from the tumor margin) were obtained from 88 patients with primary gastric cancer who underwent radical gastrectomy at Zhongshan Hospital, Fudan University (Shanghai, China) during the period from May 2007 to February 2008. All tissue samples were immediately processed after surgical removal. Diagnosis and grading were histologically confirmed by two experienced pathologists. Patients were followed until August 2013. The mean follow-up time was 35 months (1–75 months). The clinical characteristics of the patients are summarized in Table 1. This study was approved by the Ethics Committee of Fudan University. Written consent was obtained from all patients.

**Table 1:** Correlation of UPS22 expression with clinical characteristics of patients with gastric cancer

Characteristics	n	USP22-positive (%)	USP22-negative (%)	*P*-value
Gender				0.249
Male	65	53 (81.5)	12 (18.5)	
Female	23	16 (69.6)	7 (30.4)	
Age				0.338
< 60	29	21 (72.4)	8 (27.6)	
≥ 60	59	48 (81.4)	11 (18.6)	
Tumor diameter				1.000
< 5 cm	29	23 (79.5)	6 (20.5)	
≥ 5 cm	59	46 (78.0)	13 (22.0)	
Lesion site				0.101
Upper third	18	17 (94.4)	1 (5.6)	
Middle third	28	19 (67.9)	9 (32.1)	
Lower third	42	33 (78.5)	9 (21.5)	
T stage				0.019
T_1–2_	12	6 (50)	6 (50)	
T_3–4_	76	63 (82.9)	13 (17.1)	
N stage				1.000
N_0_	20	16 (80.0)	4 (20.0)	
N_1–3_	68	53 (77.9)	15 (22.1)	
M stage				1.000
M_0_	85	66 (77.6)	19 (22.4)	
M_1_	3	3 (100)	0 (0)	
Disease stage				0.995
I–II	37	29 (78.4)	8 (21.6)	
III–IV	51	40 (78.4)	11 (21.6)	
Histologic grade				1.000
II	26	21 (80.8)	5 (19.2)	
III	62	48 (77.4)	14 (22.6)	

To validate the differential expression of USP22 in gastric cancer tissue compared with normal tissue, we examined the expression of USP22 in 90 samples from The Cancer Genome Atlas (TCGA) database. The samples included 19 samples of normal gastric tissue, 65 samples of gastric adenocarcinoma tissue, and 6 samples of gastrointestinal stromal tumor tissue.

### Immunohistochemical staining with tissue microarray

Tissue microarrays containing 88 pairs of gastric cancer tissue and adjacent normal tissue samples were constructed by Shanghai Outdo Biotech (Shanghai, China). USP22 expression was detected using immunohistochemical (IHC) staining. Briefly, the paraffin-embedded sections (4-µm thick) were dewaxed in xylene and dehydrated in ethanol, followed by incubation with 3% H_2_O_2_ for 30 min. After antigen retrieval, the sections were blocked with 10% normal goat serum, then incubated with USP22-specific primary antibody (Abcam, Cambridge, UK) overnight at 4 °C. Detection of the antigen–antibody complex was performed using a secondary antibody and a DAB detection kit (Maxim Biotechnologies, Fuzhou, Fujian, China). The results were blindly scored (0–100%) under an Olympus microscope (Japan) by two independent pathologists. Five randomly selected fields were scored for each section. Staining intensity was quantified as follows: 0, no staining; 1, yellow; 2, brown; 3, dark brown. The percentages of positive cells were graded as follows: 0, < 5%; 1, 5–24%; 2, 25–49%; 3, 50–74%; 4, > 75%. The IHC score for each specimen was calculated as the product of staining intensity and grade and classified as negative (0–2) or positive (≥ 3).

### Cell lines and cell culture

The normal human gastric epithelial cell line GES-1 and human gastric cancer cell lines MGC803, SGC7901, AGS, and MKN45 were purchased from The Cell Bank of Type Culture Collection of the Chinese Academy of Sciences (China) and maintained in RPMI1640 medium (Gibco, Thermo Fisher Scientific, Waltham, MA, USA) supplemented with 10% fetal bovine serum (FBS), 100 U/mL penicillin, and 100 μg/mL streptomycin. Human lung cancer cell line H1229 and human pancreatic cancer cell line PANC1 were purchased from Shanghai Institute for Biological Science at the Chinese Academy of Science (Shanghai, China) and maintained in DMEM (Gibco) supplemented with 10% FBS. All cells were maintained in a humidified atmosphere of 5% CO_2_ at 37 °C.

### Construction of lentiviral expression vectors and transfection

For the overexpression of USP22, human USP22 cDNA was cloned into a lentiviral vector GV208 by Genechem (Shanghai, China). SGC7901 cells were transfected with the expression vector or the empty vector to establish stable USP22-overexpressing cells and control cells by puromycin selection (2 μg/mL). USP22-silencing and control AGS cells were established using lentiviral vectors expressing short hairpin RNA (shRNA) against USP22 or scrambled shRNA. shSOS1 was cloned into a lenti-U6-CMV-mcherry-puro vector by iCARTab BioMed (Suzhou, Jiangsu, China) and transduced into SGC7901 cells.

### Animals and treatment

All animal care and experimental procedures were approved by the Ethical Committee of Fudan University. All animal experiments were performed according to the Guidelines for Proper Conduct of Animal Experiments at Fudan University. BALB/c male nude mice (6–8-weeks old) were purchased from Changzhou Cavens Lab Animal Co. Ltd. (Jiangsu, China) and maintained under specific pathogen-free conditions.

To examine the oncogenic role of USP22, mice were randomly divided into 4 groups (n = 8/group). Control SGC7901 cells, USP22-overexpressing SGC7901 cells, control AGS cells, or USP22-silencing AGS cells (1 × 10^7^ cells in 0.1 mL serum-free medium) were inoculated subcutaneously into the right armpit of each mouse. To examine the role of SOS1, mice were randomly divided into 3 groups (n = 8/group). SGC7901 cells transfected with empty vector, USP22-expressing vector, or USP22-expressing vector + shSOS1-expressing vector were inoculated subcutaneously into the right armpit of each mouse.

Tumor formation was monitored every other day. Tumor volume (V) was measured twice a week after tumor formation using a digital caliper and calculated as ½ × length × width^2^. Mice were sacrificed at 30 days after inoculation. Tumors were immediately removed, weighed, and stored at − 80 °C.

### MTT assay

USP22-overexpressing SGC7901 cells, USP22-silencing AGS cells, or corresponding control cells were plated in a 96-well plate at a density of 5 × 10^4^ cells/well and cultured overnight. At 24, 48, or 72 h after incubation, the medium was removed, followed by the addition of 200 μL of serum-free medium and 20 μL of MTT (5 mg/L) to each well. After 4 h of incubation at 37 °C, the MTT-containing medium was removed, and 150 μL of dimethyl sulfoxide was added to each well. Absorbance values were measured using a plate reader (BioTek, Winooski, VT, USA) at a wavelength of 490 nm.

### Cell migration and invasion assays

Cell migration and invasion were measured with the Transwell chamber assay. For the migration assay, 1 × 10^5^ cells were seeded in the upper chamber of the Transwell device (8-μm pore size). For the invasion assay, cells were loaded into a matrigel-coated upper chamber filled with serum-free RPMI1640 medium. The lower chamber was filled with 500 μL RPMI1640 containing 20% FBS. After incubation at 37 °C for 24 h, nonmigrating or non-invading cells remaining in the upper chamber were removed with a cotton swab. The migrating or invading cells adhering to the lower surface were fixed and stained with crystal violet (0.1%). The stained cells were counted in 3 randomly selected fields under an inverted light microscope (Olympus) at 200× magnification. Images were acquired using a Leica camera (Wetzlar, Germany).

### TUNEL assay

Cell apoptosis was evaluated using a TUNEL reaction-based in situ cell death detection kit (Keygentec, Nanjing, China), according to the manufacturer’s instructions. The number of TUNEL-positive cells (purple) and the total number of cells were counted in 4 randomly selected fields under a Leica microscope at 400× magnification by two independent pathologists in a blinded manner. The percentage of apoptosis (%) was calculated as the number of TUNEL-positive cells, divided by the total number of cells × 100%. Representative images were captured using a Leica camera.

### Flow cytometry analysis

For cell-cycle analysis, cells (5 × 10^5^) were harvested and fixed with cold 70% ethanol overnight, digested with RNase A (Promega, Madison, WI, USA) at 37 °C for 30 min, and stained with propidium iodide (Invitrogen) at 4 °C in the dark for 30 min. Cell cycle phases were characterized using a FACS Calibur flow cytometer (BD Biosciences, San Jose, CA, USA) at 488 nm.

### Quantitative real-time polymerase chain reaction (qRT-PCR)

Total RNA was isolated using Trizol Reagent (Invitrogen, Carlsbad, CA, USA), according to the manufacturer’s instructions. One microgram of RNA was reverse-transcribed into cDNA using M-MLV reverse transcriptase (Promega), according to the manufacturer’s protocol. Amplification was performed using a SYBR master mixture (Takara, Japan) and gene-specific primers (Table 2) on a qRT-PCR device (Takara). GAPDH was used as an internal control.

**Table 2:** Primers for quantitative real-time PCR

Gene	Forward (5′–3′)	Reverse (5′–3′)
GAPDH	CATCTTCTTTTGCGTCGCCA	CCCCCTCTTGAATTTTAAAGGATGT
SOS1	CTGCTTCTGGTGCTTCCAGT	ATGCACTTAGAATTTTTGCACCT
ANKRD30BL	AACACCTGACACGGCTGAAA	CAGACAGCGAACAGTAGCCC
JAKMIP1	CTACGTCCGCTGCTGCT	CCTTGGGTCAGAGTGCTGAG
CDH18	GAGCTCTGGGAAAGTCCCCT	GGACTTGCCCTAACTGGTGG
GUSBP1	ACCTGTAGCCCAGCAAAGTT	ACCCTCCGAGTCTTTCCCAA
FAM189A2	AGAGCCCTGAGCCTGAGATT	ACTCTCCCTTCAAGTTGCTGA
C2orf61	AGCTTTCTCCGGGGCAATAC	GCCCATGAGGCCCTACATTA
IKZF1	GTTCTCGTCGCACATAACGC	CGGCAGTCCTTGTGCTTTTC
TTC34	CAAGAAGGGAGACGTGCCAG	CAGACAGCGAACAGTAGCCC
LINC01057	TTCGGTGACCATGTAGCCTG	ATCCACGTGGTGGGAATCAG
IPO9	ACTGGTGTGCCCAATCAGAG	GGAGCGCACTTTGCTTATCG
SYT15	TGTCCAGCAAGACCATCACC	GTCCCCCACTAGGGTCTCAT
HS3ST2	CCCATGTAGTCCTTGCCCTC	GTTCTCGTCGCACATAACGC
LOC440434	GCTGCATGACTACATTGGGG	CCAGCCTGGATAGAGTGACAA
MYO1F	CTAAGTGCCCAACAAGGACG	TGGTCACATATGAATGCAAGGC
PSG7	TTCGGGCCTAGGCTCATCT	TCAATCGTGACTTGGGCTGT

### Western blot analysis

Gastric cancer cells or tissue were lysed or homogenized with lysis buffer containing phenylmethylsulfonyl fluoride on ice, followed by centrifugation at 12,000 rpm for 15 min. Protein concentrations were measured using a Braford protein quantification kit (Keygentec). Proteins were separated on 10% SDS gel and transferred to a polyvinylidene fluoride membrane (Keygentec), followed by 1 h of blocking with 5% skim milk. The membrane was then incubated with primary antibody to human USP22 (Abcam), SOS1 (Abcam), Ras, ERK, p-ERK, c-myc, PI3K, p-PI3K, AKT, p-AKT, BAD (Cell signaling Technology, Danvers, MA, USA), BCL-2 (ABclonal Technology, Woburn, MA, USA), or GAPDH (Abways Biotechnology, Shanghai, China) overnight at 4 °C, followed by 3 washes with Tris-buffered saline containing 0.1% Tween 20 (TBST). The membrane was then incubated with horseradish peroxidase-conjugated secondary antibody (Beyotime, Shanghai, China) for 1 h at room temperature. After an additional three washes with TBST, the protein bands were visualized using an enhanced chemiluminescence reagent (Keygentec) and analyzed using a Gel Doc 2000 device (Bio-Rad, Hercules, CA, USA).

### Chromatin immunoprecipitation (ChIP)

ChIP was performed using a ChIP assay Kit (Beyotime) according to the manufacturer’s protocol. Briefly, formaldehyde cross-linked chromatin was obtained from control or USP22-overexpressing SGC7901 cells. Cross-linked chromatin was immunoprecipitated with primary antibody against USP22 (Abcam) overnight at 4 °C. Normal rabbit IgG was used as a negative control. Immunoprecipitated DNA was sequenced by BGI (Beijing, China). The raw sequencing data were analyzed using the MAnorm method.

### Statistical analysis

Statistical analyses were carried out using SPSS software (Version 23.0; SPSS Inc., Chicago, IL, USA). Data are presented as mean ± standard deviation. Comparisons among groups were conducted using analysis of variance (ANOVA) and pair-wise comparisons. Differences between categorical variables were compared using Fisher’s exact test and Pearson Chi-square test. The association between USP22 expression and variables of interest was analyzed by multivariate Cox proportional hazards analysis. A value of *P* < 0.05 was considered statistically significant.

## Results

### USP22 is overexpressed in human gastric cancer tissue and positively correlated with local tumor (T) stage

**Fig. 1:**
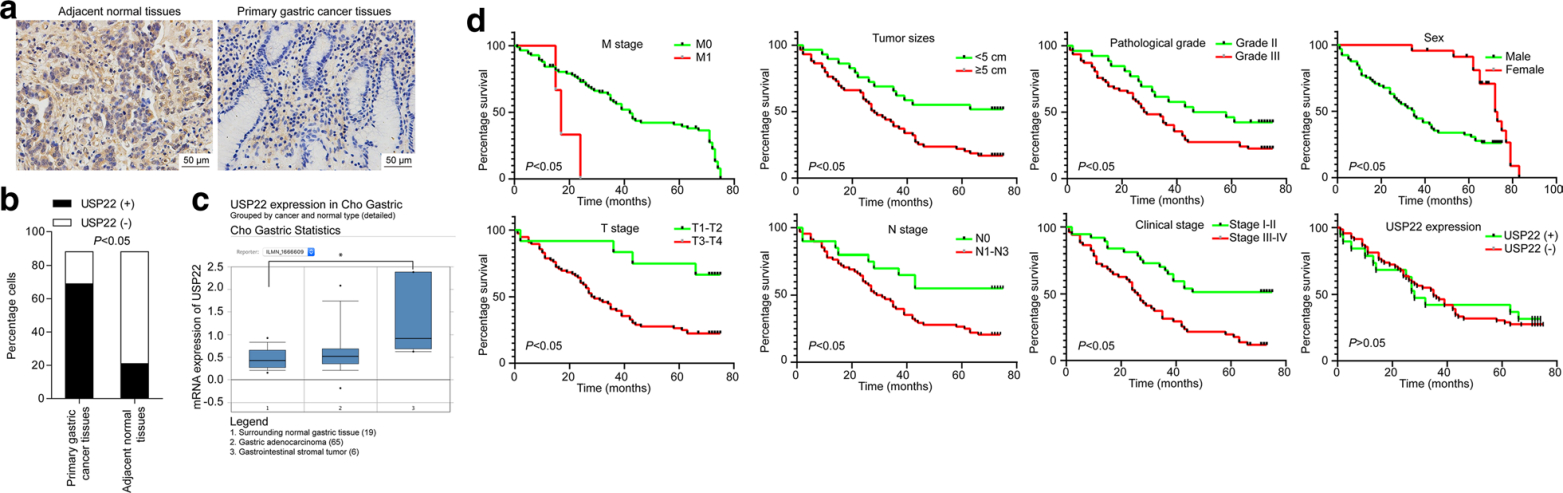
Ubiquitin-specific peptide 22 (USP22) was upregulated in gastric cancer tissue and correlated with T stage in patients. **a** Immunohistochemical staining of USP22 expression. The study included 88 samples of paired primary gastric cancer tissue (left) and adjacent normal tissue (right). Representative images are shown. Magnification ×100. **b** Quantification of **a**. **c** The mRNA expression of USP22 in 90 samples from The Cancer Genome Atlas database, including 19 samples of normal gastric tissue, 65 samples of gastric adenocarcinoma tissue, and 6 samples of gastrointestinal stromal tumor tissue. **d** Kaplan–Meier analysis of survival time in patients with gastric cancer (n = 88)

To explore the involvement of USP22 in gastric cancer, we examined the protein expression of USP22 in 88 samples of gastric cancer tissue and paired adjacent normal tissue from patients with primary gastric cancer. The results of IHC staining showed that USP22 was predominantly expressed in cancer tissue, rather than normal tissue [78.4% (69/88) vs. 23.9% (21/88), χ^2^ = 52.391, *P* = 0.001; Fig. 1a, b]. To further validate the overexpression of USP22 in gastric cancer, we collected the mRNA expression data from the TCGA database. As shown in Fig. 1c, compared with that in normal gastric tissue, the mRNA expression of USP22 in gastrointestinal stromal tumor samples was significantly increased, whereas the mRNA expression of USP22 in gastric adenocarcinoma tissue was slightly increased without statistical significance.

In addition, the presence of USP22 was positively correlated with T stage in patients with gastric cancer (Table 1), suggesting that USP22 expression is associated with gastric tumor growth. To further evaluate the potential prognostic values of USP22 expression and other clinical characteristics in gastric cancer, we determined the association of these variables with the overall survival of patients using Kaplan–Meier analysis and the log-rank test. As shown in Fig. 1d, the overall survival of patients was significantly correlated with gender, tumor size, histologic grade, TNM stage, and disease stage (all *P* < 0.05). Although USP22-negative patients had longer 5-year survival than UPS22-positive ones (42.10% vs. 30.4%, *P* > 0.05), we did not observe a significant difference, possibly due to a relatively small sample size. Multivariate Cox regression analysis indicated that tumor size and disease stage were independent prognostic factors for gastric cancer (Table 3).

**Table 3:** Multivariate Cox regression analysis of overall survival in patients with gastric cancer

Variable	*P*-value
Tumor size (< 5 cm vs. ≥ 5 cm)	0.026
Disease stage (I–II vs. III–IV)	0.01
T stage (T1–T2 vs. T3–T4)	0.145
N stage (N0 vs. N1–3)	0.805
Histologic stage (II vs. III)	0.183
Gender (male vs. female)	0.775

### USP22 promotes cell proliferation, migration, and invasion in gastric cancer cells

**Fig. 2:**
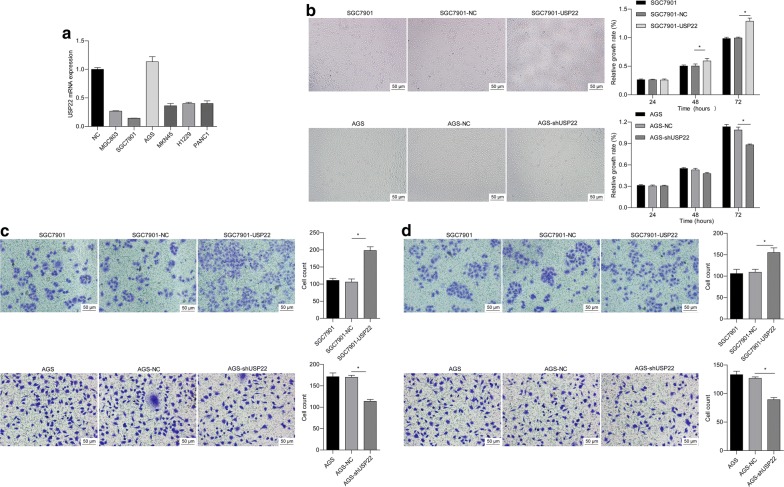
USP22 promoted cell proliferation, migration, and invasion in gastric cancer cells. **a** Quantitative real-time PCR (qRT-PCR) was performed to detect mRNA expression of USP22 in different human gastric cancer cell lines (MGC803, SGC7901, AGS, and MKN45), human lung cancer cell line (H1229), and human pancreatic cancer cell line (PANC1). The normal human gastric epithelial cell line GES-1 was used as a normal control (NC). SGC7901 and AGS cells were stably transfected with USP22- and shUSP22-expressing vectors, respectively. Cells transfected with empty vector were used as NC. **b** An MTT assay was performed to measure cell proliferation at 24, 48, and 72 h after culture. *P < 0.05 vs. NC; n = 3. **b**, **c** Cell migration and invasion were measured at 24 h after culture. *P < 0.05 vs. NC; n = 3

To select appropriate cell lines for exploring the function of USP22 in gastric cancer, we measured endogenous mRNA levels in various gastric cancer cell lines. We found that the SGC7901 and AGS cell lines had the lowest and highest levels of endogenous USP22 mRNA, respectively, compared with normal human gastric epithelial GES-1 cells (Fig. 2a). To further explore the role of USP22, we performed gain- and loss-of-function assays in SGC7901 and AGS cells, respectively. Compared with the corresponding negative control, USP22 overexpression significantly promoted cell proliferation, migration, and invasion in SGC7901 cells, whereas USP22 knockdown significantly inhibited these behaviors in AGS cells (Fig. 2b–d). These data suggest that USP22 promotes gastric cancer development in vitro.

### USP22 inhibits cell apoptosis while promoting cell cycle transition in gastric cancer cells

**Fig. 3:**
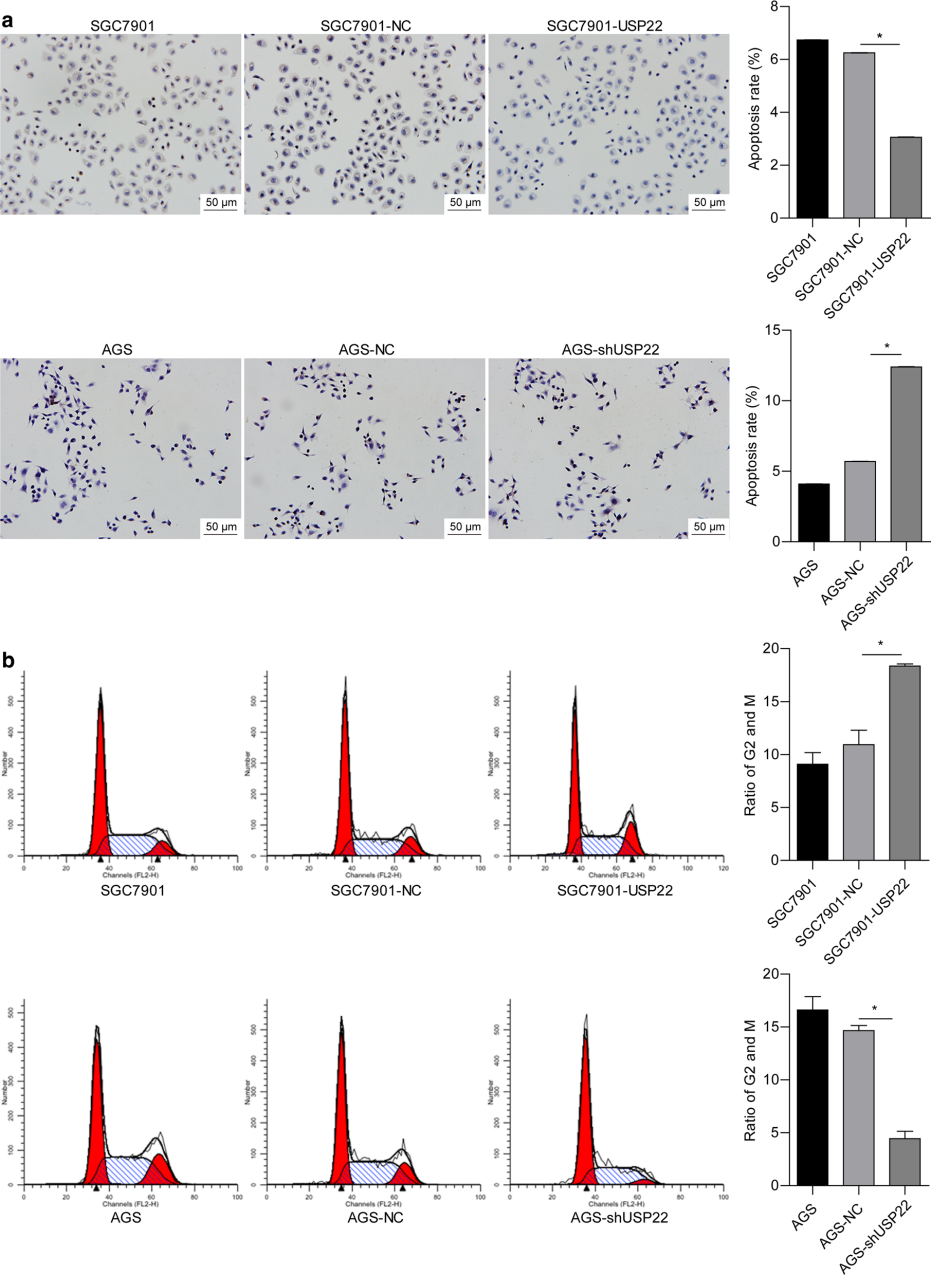
USP22 suppressed apoptosis while inducing cell cycle transition in gastric cancer cells. **a**, **b** The TUNEL assay was performed to measure the rate of apoptosis in SGC7901 (**a**) and AGS cells (**b**). **c**, **d** Cells were stained with propidium iodide. Cell cycle progression was assessed using flow cytometry. *P < 0.05 vs. NC; n = 3

To further explore the role of USP22 in gastric cancer, we examined cell apoptosis and cell cycle progression in USP22-overexpressing or -silenced gastric cancer cells. The TUNEL assay showed that USP22 overexpression significantly suppressed apoptosis in SGC7901 cells, whereas silencing enhanced apoptosis in AGS cells (Fig. 3a). In addition, USP22 overexpression induced a significant increase in the proportion of SKOV3 cells in the G2/M phase. The opposite effect was observed in USP22-deficient AGS cells (Fig. 2b). These findings suggest that USP22 accelerates gastric cancer cell proliferation, possibly by suppressing cell apoptosis and promoting the G2/M transition.

### USP22 facilitates gastric tumor growth in vivo

**Fig. 4:**
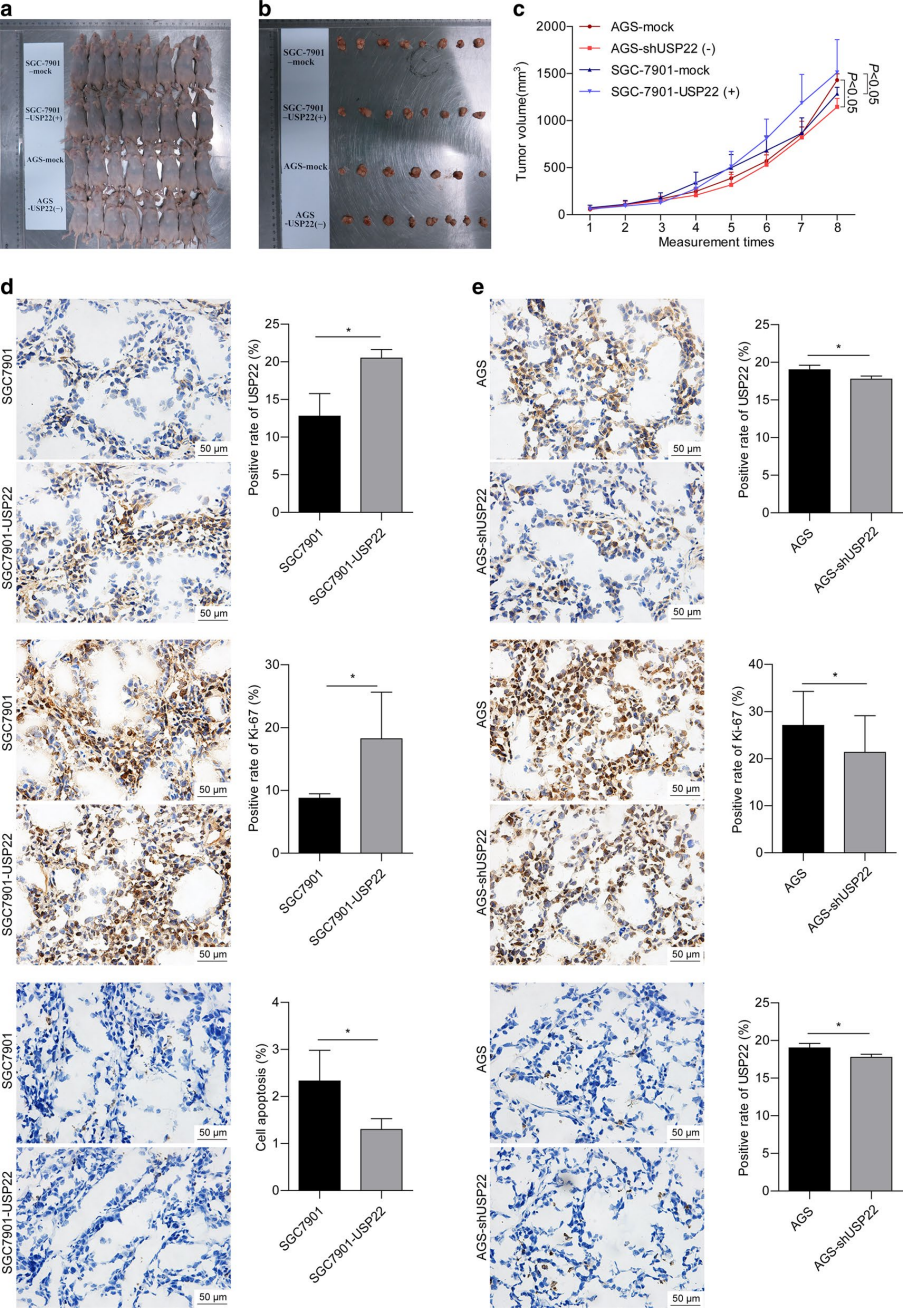
USP22 promoted tumor growth in nude mice. **a** BALB/c nude mice were randomly divided into 4 groups (n = 8/group). USP22-overexpressing SGC7901 cells, USP22-silenced AGS cells, or corresponding negative control cells were subcutaneously inoculated into the right armpit of each mouse. **b** Mice were sacrificed at 30 d after inoculation. Tumors were immediately harvested. **c** Tumor volume (V) was measured twice a week after tumor formation and calculated as ½ × length × width^2^. **d**, **e** Tumor tissue sections were subjected to immunohistochemical staining for USP22, Ki-67, and TUNEL expression. Images were acquired at magnification ×100. Representative images are shown

We used a nude mouse xenograft model to further investigate the role of USP22 in vivo. As shown in Fig. 4a–c, the mice inoculated with USP22-overexpressing SGC7901 cells had significantly greater tumor mass than those in the control group. IHC staining showed that the tumors developed from USP22-overexpressing SGC7901 cells had significantly increased USP22 and Ki-67 staining and decreased TUNEL staining, compared with the control group (Fig. 4d). The opposite effect was observed in the tumors developed from USP22-silenced AGS cells (Fig. 4e). These results suggest that USP22 promotes gastric cancer growth while inhibiting apoptosis in vivo.

### SOS1 is upregulated in USP22-overexpressing gastric cancer cells

To investigate the mechanism underlying the oncogenic role of USP22 in gastric cancer, we performed a ChIP-seq assay and found 16 differentially expressed genes (Additional file 1: Table S1) in USP22-overexpressing SGC7901 cells, compared with control cells. The results of qRT-PCR confirmed that the mRNA levels of 8 genes (SOS1, ANKRD30BL, JAKMIP1, CDH18, GUSBP1, FAM189A2, C2orf61, and IKZF1) were significantly elevated in USP22-overexpressing SGC7901 cells. Among them, we found SOS1, an activator of oncoprotein RAS [20].

**Fig. 5:**
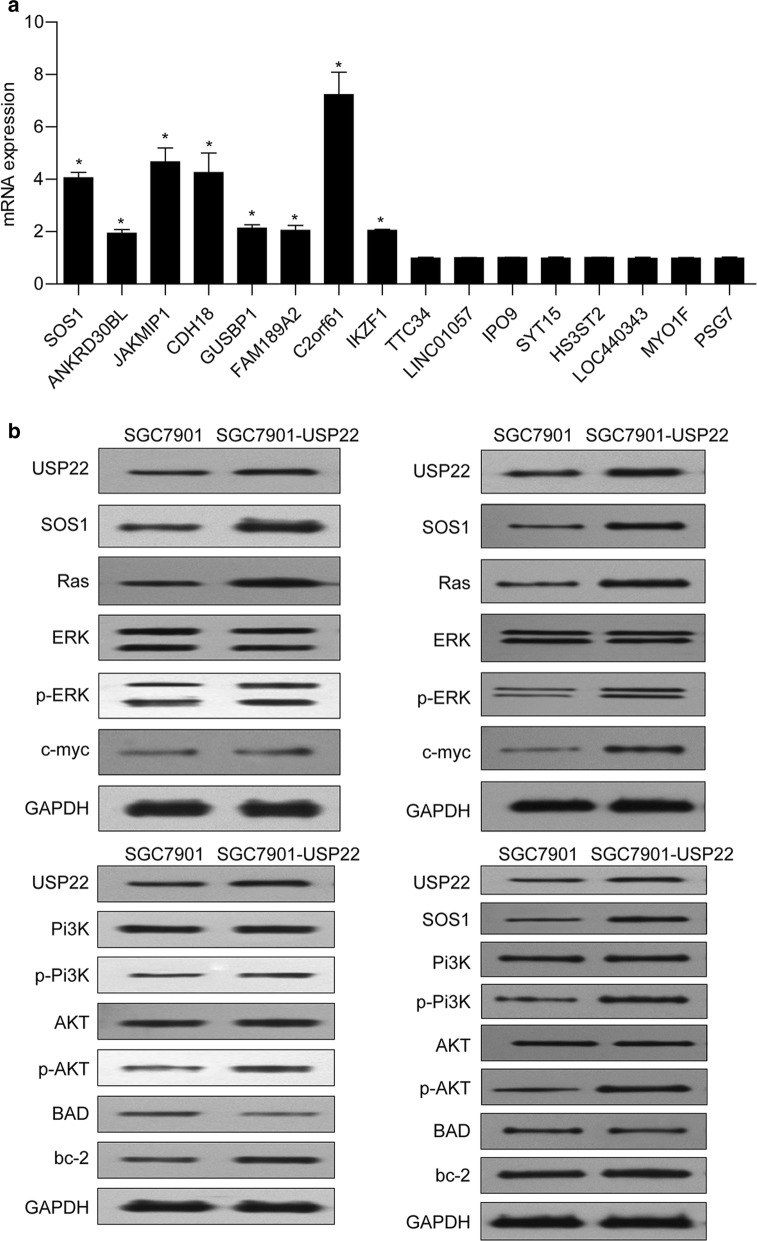
USP22 overexpression increased son of sevenless 1 (SOS1) expression and activation of the RAS/ERK and PI3K/AKT pathways. **a** USP22-overexpressing SGC7901 cells and negative control cells were subjected to a chromatin immunoprecipitation assay. qRT-PCR was performed to validate the differential expression of some genes. *P < 0.05 vs. NC; n = 3. **b** Western blot analysis was performed to determine the protein levels of USP22, SOS1, Ras, ERK, p-ERK, c-myc, PI3K, AKT, p-AKT, BAD, and bcl-2 in USP22-overexpressing SGC7901 cells and tumor tissue. GAPDH was used as an internal control

We then performed Western blot analyses to examine the activation of the RAS/ERK and RAS/PI3K/AKT pathways. The results showed that overexpression of USP22 induced considerable upregulation of SOS1 expression in SGC7901 cells, accompanied by significant upregulation of RAS, p-ERK, c-myc, p-PI3K, and p-AKT (Fig. 5b, left panel). We also noticed that overexpression of USP22 induced significant alterations in apoptosis-related BAD and bcl-2 expression. Similar results were observed in tumor tissue derived from USP22-overexpressing SGC7901 cells (Fig. 5b, right panel). These findings suggest that SOS1/RAS and downstream ERK and PI3K/AKT pathways mediate the oncogenic role of USP22 in gastric cancer.

### Knockdown of SOS1 reverses the oncogenic effects of USP22 in gastric cancer cells

**Fig. 6:**
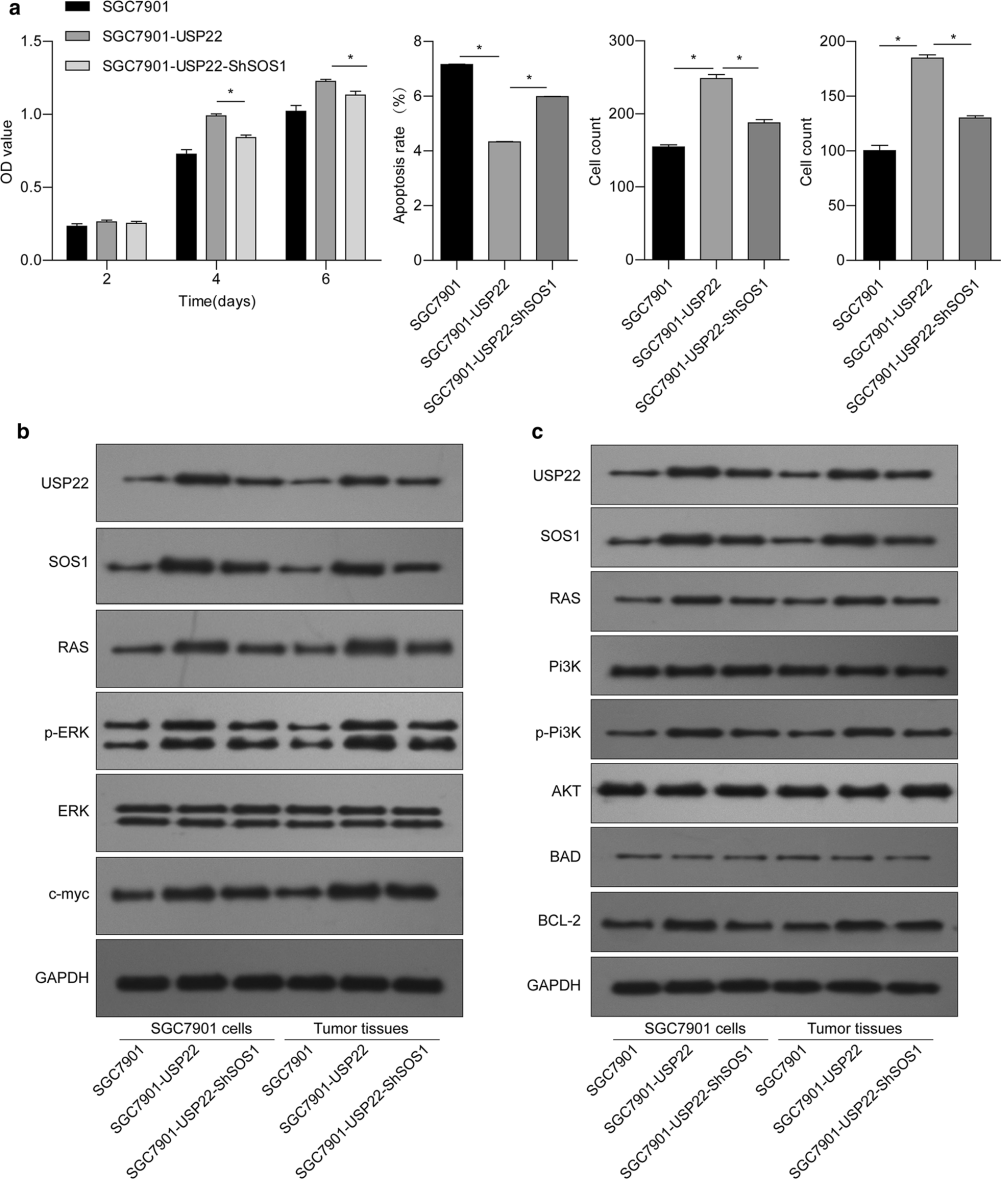
SOS1 silencing reversed USP22-induced changes in gastric cancer cell behavior and RAS signaling. USP22-overexpressing SGC7901 cells were transfected with shSOS1. **a** Cell proliferation, apoptosis, migration, and invasion were evaluated. **b**, **c** Western blot analysis was performed to measure protein levels of USP22, SOS1, Ras, ERK, p-ERK, c-myc, PI3K, AKT, p-AKT, BAD, and bcl-2 in SGC7901 cells and tumor tissue. GAPDH was used as an internal control. *P < 0.05 vs. USP22-overexpressing SGC7901 cells; n = 3

Next, we performed a loss-of-function assay to investigate whether SOS1 plays an essential role in the oncogenic effects of USP22 on gastric cancer cells. We found that the knockdown of SOS1 effectively reversed the effects of USP22 overexpression on cell proliferation, apoptosis, migration, and invasion in SGC7901 cells (Fig. 6a). Western blot analyses showed that the knockdown of SOS1 could also attenuate USP22-induced activation of the SOS1/RAS/ERK and SOS1/RAS/PI3K/AKT pathways in both SGC7901 cells and xenograft tumors (Fig. 6b, c). These results suggest that SOS1 plays an essential role in mediating the oncogenic effects of USP22 on gastric tumor growth, possibly via activating the RAS/ERK and PI3K/AKT pathways.

## Discussion

Although previous studies have extensively reported USP22 overexpression and its oncogenic role in cancer [21–23], recent studies suggest USP22 downregulation and tumor suppressor properties in some cancers [9, 10]. Clarifying the expression and activity of USP22 in gastric cancer is critical to determine whether targeting USP22 will be beneficial for patients. In this study, by assessing the expression of USP22 in 88 paired samples of primary gastric tumor tissue and adjacent normal tissue, we demonstrated that USP22 is predominantly expressed in gastric cancer tissue. This finding is consistent with previous reports [24, 25] showing upregulation of both USP22 mRNA and protein expression in patients with gastric cancer. By analyzing the mRNA expression data from the TCGA database, we found that the mRNA expression of USP22 in gastrointestinal stromal tumor samples was significantly increased compared with that in normal gastric tissue. However, the mRNA expression of USP22 in gastric adenocarcinoma tissue was slightly increased without statistical significance. This discrepancy may be related to ethnicity and the extent of malignancy. We also evaluated the effects of USP22 overexpression and knockdown on the malignant behaviors of gastric cancer cells both in vitro and in vivo. Our results indicate that USP22 promotes cell proliferation, migration, invasion, and cell cycle transition, while inhibiting apoptosis in gastric cancer cells. A xenograft mouse model showed consistent results. Despite numerous previous studies on the roles of USP22 in cancer, the effectors and signaling pathways downstream of USP22 are not fully understood. In this study, using a ChIP assay, we demonstrated, for the first time, that the overexpression of USP22 induced the upregulation of RAS activator SOS1 in SGC7901 cells. Furthermore, SOS1 upregulation in USP22-overexrpessing gastric cancer cells and xenograft tumor tissue is accompanied by activation of the RAS/ERK and RAS/PI3K/AKT pathways, which is attenuated by SOS1 silencing. These results suggest that SOS1/RAS signaling mediates the oncogenic role of USP22 in gastric cancer. Our findings identify the USP22/SOS1/RAS axis as a novel and promising therapeutic target in gastric cancer.

A recent meta-analysis of 9 studies that included 2876 cases reported that high expression of USP22 predicts poor overall survival and advanced clinicopathological characteristics in solid tumors [26]. Yang et al. reported that the co-upregulation of USP22 and oncogene BMI-1 correlates with advanced TNM stage and shorter disease-specific survival [6] in gastric cancer. Liu et al. showed that high expression of USP22 positively correlates with gastric cancer growth and metastasis, serving as an independent prognostic predictor in patients [25]. In this study, we detected a positive correlation between USP22 expression and T stage in 88 patients with gastric cancer, suggesting that USP22 expression is associated with gastric tumor growth. Although USP22-negative patients had longer 5-year survival than UPS22-positive patients, no significant difference was observed. These inconsistencies with previous studies may be due to the relatively small sample size in this study.

Among the seven cell lines investigated in our study, the SGC7901 and AGS cell lines had the lowest and highest endogenous USP22 mRNA levels, respectively, compared with a normal human gastric epithelial cell line (GES-1). The observed variation in USP22 expression among cell lines may be related to the extent of differentiation and malignancy. Therefore, we explored the roles of USP22 in USP22-overexpressing SGC7901 cells and USP22-silenced AGS cells. USP22 promotes cell proliferation, migration, and invasion in multiple cancers, including glioma, lung adenocarcinoma, thyroid carcinoma, colorectal cancer, and gastric cancer [27–31]. Consistent with these findings, our data demonstrate that USP22 promotes gastric cancer cell proliferation, migration, and invasion in vitro and enhances tumor growth in vivo. Furthermore, the results of flow cytometry showed that USP22 overexpression increases the proportion of gastric cancer cells in the G2/M phase, whereas USP22 silencing reduces this proportion. Similarly, in pancreatic ductal adenocarcinoma cells, USP22 promotes the G1/S transition and proliferation by upregulating the expression of FoxM1 [8], a key regulator of the G1/S and G2/M cell cycle transitions [32]. In addition, USP22-mediated de-ubiquitination protects CCNB1, another master regulator of the G2/M transition, from degradation [33]. These findings indicate that USP22 may promote gastric cancer cell proliferation by accelerating the G2/M progression. However, the underlying mechanisms need further investigation.

As a deubiquitinating enzyme, USP22 exerts its functions via interaction with different substrates, but its downstream effectors remain to be conclusively identified. In this study, the results of a ChIP assay allowed us to demonstrate for the first time that USP22 may interact with RAS activator SOS1 in gastric cancer. SOS1 activates RAS by catalyzing the exchange of GDP for GTP, thus triggering the oncogenic RAS/ERK and RAS/PI3K/AKT pathways, both of which are essential for cell survival [34]. Indeed, USP22 overexpression induces significant elevations in protein levels of SOS1, RAS, p-ERK, p-PI3K, and p-AKT in both SGC7901 cells and tumors. C-myc plays an essential role in gastric cancer cell proliferation [35]. The activation of RAS/ERK signaling promotes c-myc mRNA expression and stabilizes c-myc, whereas inhibition of this pathway downregulates c-myc expression and thus inhibits cancer cell growth [36]. In this study, we found that USP22 overexpression induces c-myc upregulation in both SGC7901 cells and tumor tissue, consistent with previous reports. In addition, active AKT can phosphorylate Bcl-2-associated death protein (BAD) on ser-136 and facilitate its dissociation from the Bcl-2/Bcl-X complex, thereby suppressing the apoptosis-inducing function of BAD [37]. This may explain changes in BAD and bcl-2 expression in response to USP22 overexpression.

To investigate whether SOS1 plays an essential role in mediating the tumorigenic roles of USP22 in gastric cancer, we silenced its expression in USP22-overexpressing SGC7901 cells. We found that SOS1 silencing effectively reversed USP22-induced changes in cell proliferation, migration, invasion, and apoptosis in vitro. SOS1 silencing also attenuated the USP22-induced activation of RAS/ERK and PI3K/AKT signaling both in vitro and in vivo. The downstream effectors involved in cell proliferation (c-myc) and apoptosis (BAD and bcl-2) exhibit consistent changes. These data suggest that USP22 regulates gastric cancer cell growth and survival in an SOS1-dependent manner. Direct inhibition of RAS in cancer therapy is challenging and has failed so far due to the high intracellular concentration of GTP and its picomolar affinity for RAS [38]. It has been reported that treatment with SOS1 inhibitors results in complete inhibition of the RAS/ERK pathway in cells with wild-type KRAS and 50% inhibition in cells with mutant KRAS cells [20]. Consistently, our study also suggests that targeting SOS1 is a promising strategy in RAS-driven tumors.

## Conclusions

In this study, we demonstrate that USP22 is highly expressed in gastric cancer tissue, compared with adjacent normal tissue. USP22 acts as an oncogene in gastric cancer, as evidenced by the enhancement of aggressive cancer cell behavior induced by USP22 overexpression. USP22 overexpression in gastric cancer cells induces the upregulation of SOS1 and activation of the RAS/ERK and PI3K/AKT pathways. The oncogenic role of USP22 in gastric cancer relies on SOS1 expression. Targeting the USP22/SOS1/RAS axis represents a promising strategy in gastric cancer therapy.

## Supplementary information

**Additional file 1: Table S1.** Genes with differential expression in USP22-overexpressing SGC7901 vs. control cells.

## Data Availability

The datasets generated and analyzed during the current study are available from the corresponding author on reasonable request.

## Abbreviations

USP22: Ubiquitin-specific peptide 22
SOS1: Son of sevenless 1
GEF: Guanine nucleotide exchange factor
ERK: Extracellular signal-regulated kinase
PI3K: Phosphatidylinositol 3-kinase
IHC: Immunohistochemical
shRNA: Short hairpin RNA
FBS: Fetal bovine serum
ChIP: Chromatin immunoprecipitation
qRT-PCR: Quantitative real-time polymerase chain reaction
TBST: Tris-buffered saline containing 0.1% Tween 20
BAD: Bcl-2-associated death protein
