# Supplementary material for: Ubiquitin-specific peptide 22 acts as an oncogene in gastric cancer in a son of sevenless 1-dependent manner
Source: Cancer Cell Int. 2020 Feb 10;20:45. doi: 10.1186/s12935-020-1137-y (PMC7011508; doi:10.1186/s12935-020-1137-y)
Supplement: Supplementary file 1 — Additional file 1: Table S1. Genes with differential expression in USP22-overexpressing SGC7901 vs. control cells. [file 12935_2020_1137_MOESM1_ESM.doc]

**Table S1.** Genes with differential expression in USP22-overexpressing SGC7901 vs. control cells

| ID | Gene |
| --- | --- |
| 100287898 | TTC34 |
| 101928079 | LINC01057 |
| 55705 | IPO9 |
| 83849 | SYT15 |
| 9956 | HS3ST2 |
| 440434 | LOC440434 |
| 4542 | MYO1F |
| 5676 | PSG7 |
| 6654 | SOS1 |
| 285051 | C2orf61 |
| 554226 | ANKRD30BL |
| 152789 | JAKMIP1 |
| 1016 | CDH18 |
| 728411 | GUSBP1 |
| 10320 | IKZF1 |
| 9413 | FAM189A2 |
